# Molecular identification of *Salmonella* Typhimurium from village chickens based on *invA* and *spvC* genes

**DOI:** 10.14202/vetworld.2020.764-767

**Published:** 2020-04-23

**Authors:** Mwanaisha Mkangara, Ernest R. Mbega, Musa Chacha

**Affiliations:** 1 Department of Sustainable Agriculture and Biodiversity and Ecosystems Management, Nelson Mandela African Institution of Science and Technology, Arusha, Tanzania; 2 Department of Science and Laboratory Technology, Dar es Salaam Institute of Technology, Dar es Salaam, Tanzania

**Keywords:** invasive gene A, polymerase chain reaction, *Salmonella* plasmid virulence gene, *Salmonella* Typhimurium, sequencing

## Abstract

**Aim:**

This study aimed to identify *Salmonella enterica* serovars by polymerase chain reaction (PCR) based on virulence genes invasion A (*invA*) and *Salmonella* plasmid virulence C (*spvC*).

**Materials and Methods:**

DNA extraction of eight bacteria isolates was done using the PowerSoil^®^ DNA Isolation Kit. The amplification of *invA* and *spvC* genes was done using conventional PCR. The positive PCR products were purified using the GeneJET Purification Kit and then sequenced using ABI 3730 XL automated genetic analyzer. The sequences obtained were compared for similarities with other *Salmonella* serovars deposited on the NCBI GenBank using BLASTN.

**Results:**

Four out of eight samples were amplified by primers FS139/RS141 that target *invA* gene with products of about 284 bp, and three out of four of the same *invA* positive samples were also amplified by primers FSPV-1/RSPV-2 targeting *spvC* with a product of about 571 bp. One sample was not amplified by primers FSPV-1/RSPV-2 as it lacked virulence plasmid. Analysis of sequences indicated 100% homology with closely related serovars of *S*. *enterica* subspecies enterica serovar Typhimurium.

**Conclusion:**

*Salmonella* Typhimurium that contained *invA* and *spvC* genes are pathogenic and virulent strains.

## Introduction

*Salmonella enterica* serovar Typhimurium (*Salmonella* Typhimurium) is a motile Gram-negative bacterium in family *Enterobacteriaceae* [1]. The bacterium is one among serovars with broad host range responsible for gastroenteritis in chickens but rarely induce systemic infection [2]. In young chickens, S. Typhimurium results in severe inflammation and intestinal pathology [3]. However, in some cases, the pathogen does not cause noticeable clinical symptoms in older chickens [4,5]. Under these circumstances, the older chickens become carriers of *S*. Typhimurium, which colonizes the gut with persistent shedding of bacteria that contaminate different environments [6-9]. The carrier chickens with salmonellosis are among sources of contaminated chicken products in the abattoirs with the significance of forming biofilm layers in equipment involved in the value chain [10]. The biofilm contributes to the persistence of *Salmonella* in different biotic and abiotic surfaces and protects the bacterium against antibiotics and disinfectants [11]. On the other side, salmonellosis is zoonotic; therefore, chicken products contaminated with *S*. Typhimurium increase number of gastroenteritis infections to humans who are the most affected than any other group worldwide [12].

There are different methods of identifying pathogenic microbes from clinical samples [13]. The best choices consider techniques which are cost-effective, accurate, rapid, and easier to use to improve diagnostic testing with the outcome of right treatments in a shorter time [14]. The conventional microbiological methods, including culture using selective medium, microscopy, Gram staining, and biochemical tests are labor-intensive, time-consuming with low specificity [15,16]. In contrary to these methods, the polymerase chain reaction (PCR) is the confident and rapid technique of detecting clinical samples with higher specificity and sensitivity [17,18]. Therefore, the PCR methods qualify for the identification of any number of pathogens using specific primers that amplify the minimal part of the genome. The invasion A (*invA*) is an invasion-related gene located in *Salmonella* pathogenicity island-1 [19]. The gene is responsible for the invasion of epithelial cells and the induction of macrophage apoptosis [20]. The *invA* gene is a conserved virulence gene in nearly all *Salmonella* species; therefore, the right candidate for the detection of *Salmonella* using different PCR methods [20]. The study also selected the *Salmonella* plasmid virulence C (*spvC)* gene, which is of virulence plasmid for detecting strains associated with non-typhoid bacteremia [21]. *Salmonella* has plasmids with a highly conserved locus known as *spv* operon. The operon consists of five genes *spvRABCD* essential for *Salmonella* virulence [22]. However, not all *Salmonella* serovars consist of *spv* genes [23].

Therefore, the present study used conventional PCR for the detection of virulence genes *invA* and *spvC* in *Salmonella* spp. isolated from fecal samples of chickens.

## Materials and Methods

### Ethical approval

This study got approval from Kibong’oto Infectious Diseases Hospital, Nelson Mandela African Institution of Science and Technology (NM AIST) and Centre for Educational Development in Health, Health Research Committee with approval number KNCHREC006.

### Bacterial isolates

Eight bacterial isolates used in this study were among 54 bacteria isolates obtained from fecal samples of village chickens in Tengeru ward, Arusha, Tanzania in June 2019. Out of 54 isolates, 46 were biochemically identified to be *Salmonella* Gallinarum (in the other study). The remaining eight isolates were non-*S*. Gallinarum subjected to molecular identification using PCR.

### DNA extraction

The DNA extraction was done using the PowerSoil^®^ DNA Isolation kit (MO BIO Laboratories, Inc.) according to the manufacturer’s instruction. The quantity and purity of DNA were assessed using Nanodrop, and quality of DNA was checked on a gel of 1.5% (w/v) agarose and visualized using ultraviolet (UV)–visible spectrophotometer (Cole-Parmer UV Transilluminator). The DNA was stored at −20°C until use.

### PCR

The PCR amplification was carried out with a 25 μL amplification mixture consisting of 12.5 μL master mix (OneTaq^®^ Quick-Load^®^ 2×MM w/Std. buffer, BioLabs, New England), 0.5 μL of 10 μM each primer (Table-1), [24,25] 8.5 μL of nuclease-free water, and 3.0 μL DNA template. Amplification was conducted in a thermocycler (C-1000 Touch^™^ thermocycler). The cycle conditions consisted an initial denaturation 94°C for 1 min followed by 35cycles of denaturation at 94°C for 60 s, annealing at 64°C for 30 s, and elongation at 72°C for 30 s with 7min final extension period at 72°C. The amplified products were separated by gel electrophoresis containing 1.5%w/v agarose (SRL, India) stained with ethidium bromide (0.5 μg/mL) and detected by gel documentation system (UVP, UK). The amplification cycles for *spvC* gene were similar to *invA* gene except the annealing temperature for *spvC* gene was 58°C instead of 64°C used for *invA*.

**Table 1:** Primer pair used to amplify *Salmonella* isolates.

Gene	Primers	Oligonucleotides (5’- 3’)	Annealing temperature	Length (bp)	Reference
*invA*	S139	GTG AAA TTA TCG CCA CGT TCG GGC AA	64°C	284	[24]
	S141	TCATCGCACCGTCAAAGGAACC			
*spvC*	SPV-1	ACTCCTTGCACAACCAAATGCGGA	58°C	571	[25]
	SPV-2	TGTCTTCTGCATTTCGCCACCATCA			

*invA*=Invasion A

### Nucleotide sequencing of *invA* and *spvC* genes

The positive PCR products were purified using the GeneJET Purification Kit (Thermo Fisher Scientific, MA, USA); then, the pure products were sequenced using both forward and reverse primers (Table-1).

A gene in an ABI 3730 XL automated sequencer (Applied Biosystems, MA, USA) was in a custom sequencing facility of Mbeya Referral Hospital, Tanzania. The sequences obtained were analyzed, and homology searches were conducted using the BLAST algorithm (www.ncbi.nlm.nih.gov/BLAST).

## Results

Four samples were amplified by *invA* gene. Three out of four of the same *invA* positive samples were also amplified by *spvC* gene (Table-1). The amplified samples were 100% similar with *S. enterica* serovar Typhimurium strain number 14028 with accession number CP034479.1 and *S. enterica* subspecies enterica serovar Typhimurium strain number 14028 with accession number CP034480.1

**Figure-1:**
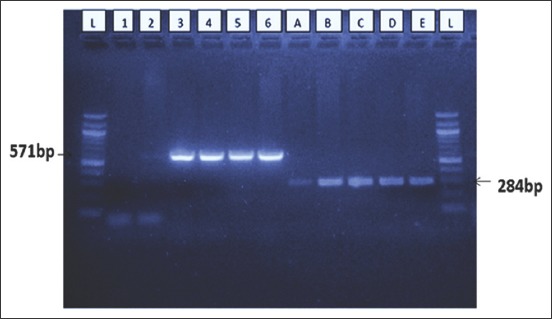
The polymerase chain reaction amplification for *invA* (284bp) and *spvC* (571bp) virulence gene of *Salmonella* spp. Lis the molecular weight marker (1000bp), 1 is a negative control, 2, 3, 4, and 5 are samples, while 6 is positive control. The B, C, D, and E are samples and A is a positive control.

The positive PCR reactions in this study were observed by agarose jelly with ~ 284 and ~571bp products for *invA* and *spvC* genes, respectively (Figure-1). The nucleotide sequence of the *invA* gene of *S*. Typhimurium strain obtained in this study was analyzed and 284bp sequences deposited with NCBI under GenBank accession number MK204827.

## Discussion

In the present study, the detection of *Salmonella* spp. by conventional PCR was done using pair of primers (FS139-RS141) specific for the *invA* gene and FSPV-1 and RSPV-2 for the *spvC* gene. The selection of these genes was made based on those associated with virulence in *Salmonella* spp. [26]. The *invA* gene of *Salmonella* contains sequences unique to this genus and has been approved as a suitable PCR target with the potential diagnostic application [20]. This gene encodes a protein in the inner membrane of bacteria responsible for the invasion of the epithelial cells of the host [19]. On the other side, *spvC* gene targets invasive non-typhoidal *Salmonella* spp. that induces systemic infection to chickens [27,28].

In this study, out of four samples amplified for *invA* gene, three were positive with *spvC*. Following blasting of the sequences for *invA* positive, *spvC* positive, and *spvC* negative, it was revealed that all samples were *S. enterica* serovar Typhimurium. The lack of *spvC* gene to one sample of *S*. Typhimurium is an indication that the species is non-invasive compared to invasive non-typhoidal *S*. Typhimurium which possess *spvC* gene. In support of these findings, Guiney and Fierer [29] observed that three genes represent virulence phenotype in spv locus designated as *spvRABCD* are positive transcriptional regulator *spvR* and two structural genes *spvB* and *spvC* [30]. The *spvBC* are sufficient to replace all of the spv genes, as well as the entire virulence plasmid to enable systemic infection in mice after subcutaneous inoculation [31]. However, in the absence of *spvC*, *spvB* does not have a detectable virulence phenotype [21]. From these observations, it is suggested that *spvC* gene is the primary determinant of virulence in spv locus. However, the exact mechanisms by which *spvB* and *spvC* manage to enhance virulence are still unclear [31].

It is becoming clear that the virulence of *S*. Typhimurium identified from this study is attributed by the possession of both *invA* and *spvC* genes [32,33]. *S*. Typhimurium, which lacks *spvC* gene, eliminates the ability of plasmid to confer virulence to chickens [21,34].

## Conclusion

*InvA* and *spvC* genes identified pathogenic and virulent strains of *S*. Typhimurium. The *invA* is a factor in the outer membrane of *Salmonella* spp., thus enables *S*. Typhimurium to initiate infection on the epithelial cell of chicken and induction of macrophage apoptosis. *S*. Typhimurium containing *spvC* gene is more virulent than those lacking this gene. This is because virulence plasmid with a structural gene *spvC* plays a role in the pathogenicity of *S*. Typhimurium. However, not all *Salmonella* serovars possess *spvC* gene responsible for virulence phenotype. Therefore, relying only on the *spvC* gene for the detection of *Salmonella* spp. may provide a high chance of false-positive results because some non-typhoidal *Salmonella* like *S*. Typhimurium possess *spvC* gene and others lack it.

## Authors’ Contributions

MM planned the study, collected samples, analyzed data, and developed the manuscript. ERM analyzed data, edited, and proofread the manuscript; MC analyzed data and wrote the manuscript. All authors read and approved the final manuscript.
